# Curating and sharing XAS data – Metadata and Scientific quality control

**DOI:** 10.1038/s41597-026-07966-x

**Published:** 2026-08-01

**Authors:** Abhijeet Gaur, Sebastian Paripsa, Frank Förste, Dmitry E. Doronkin, Wolfgang Malzer, Christopher Schlesiger, Birgit Kanngießer, Dirk Lützenkirchen-Hecht, Edmund Welter, Jan-Dierk Grunwaldt

**Affiliations:** 1 Institute for Chemical Technology and Polymer Chemistry, Karlsruhe Institute of Technology (KIT), Engesserstr. 20, Karlsruhe, D-76131 Germany; 2 Fk. 4, Physik, Bergische Universität Wuppertal, Gauß-Str. 20, Wuppertal, D-42097 Germany; 3 Deutsches Elektronen-Synchrotron (DESY), Notkestraße 85, Hamburg, D-22607 Germany; 4 Institute for Physics and Astronomy, Technische Universität Berlin, Hardenbergstr. 36, Berlin, D-10623 Germany; 5 Institute of Catalysis Research and Technology, Karlsruhe Institute of Technology (KIT), Hermann-von-Helmholtz-Platz 1, Eggenstein-Leopoldshafen, D-76344 Germany

## Abstract

With the growing volume of measured X-ray Absorption Spectroscopy (XAS) data and their need for machine-learning and reference purposes across different facilities, both spectral quality and documentation of the XAS measurements need to be standardized. The documentation is also important to avoid unnecessarily repeating reference measurements and therefore wasting precious beamtime. In this article, we have discussed the classification of quality control with respect to meta data and scientific quality, important for standard documentation and curation of the XAS data. As an example, we take the use case of the RefXAS database developed under the DAPHNE4NFDI project. Considering that the database is under development, the initial requirement for an XAS database is a comprehensive set of metadata fields, that enhances the interpretation of XAS spectra; thereby improving its reusability and the reproducibility. The metadata schema should be able to provide details about the sample, optimized equipment and measurement conditions thereby making the process of acquiring the data clear. The next important component is the evaluation of the quality of the spectra as raw data and the metadata set to ensure the accuracy and reliability of the data stored in the database. Quality criteria need to be formulated for automated initial screening of any uploaded data set followed by manual curation involving utilization of details of metadata provided during upload and scientific quality of the XAS data. In this way, metadata and scientific quality control for XAS data uploaded at a database will provide a well-defined protocol for curation of the data and strengthen its distribution under FAIR data principles.

## Introduction

With the advancement in XAS beamline set ups across the synchrotron facilities for time-resolved as well as spatially-resolved measurements and the availability of laboratory-based spectrometers optimized for day to day measurements, X-ray Absorption Spectroscopy (XAS) has become a standard approach for the structural characterisation of complex materials. Due to the sharp increase in the volume of XAS data, availability of curated XAS measurements along with the structured metadata ensuring high quality as well as reusability become of critical importance for the user community^1,2^.

In this work, we present and discuss the classification of quality criteria for screening of the XAS spectra, taking the RefXAS database^3^ as a use case. The RefXAS database has been developed as part of the DAPHNE4NFDI^4^ initiative to serve as a curated, open-access database of XAS spectra alongside its dedicated metadata. The database is fully functional, offering open access to the users for the XAS data download as well as upload. A structured metadata schema has been formulated according to the needs of the user community for data upload. Based on the mode of measurements, quality criteria are formulated for initial screening of any uploaded reference data followed by manual curation which involves utilization of details of metadata provided during upload and scientific evaluation of the data.

**Fig. 1:**
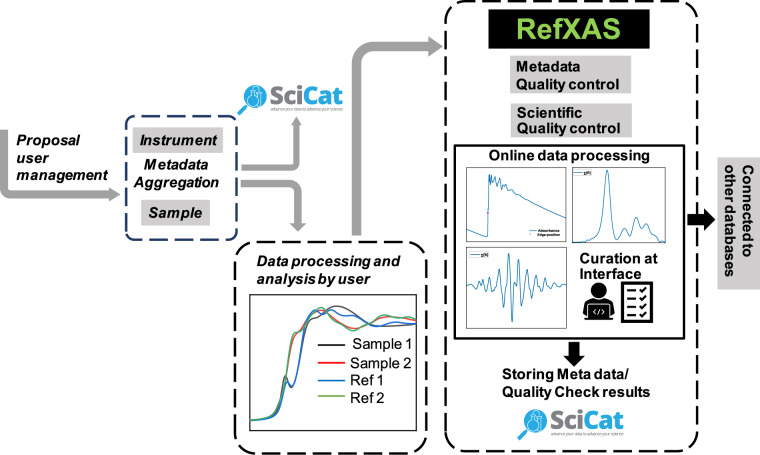
Data workflow for the *RefXAS* database. Initial aggregation of the instrument metadata and sample metadata at a Synchrotron or Laboratory based XAS facility, followed by the data analysis steps involving references, inclusion of RefXAS with the structured metadata and defined quality criteria for the XAS data along with the visualization tools. SciCat is the scientific metadata catalogue (https://www.scicatproject.org/) used for saving the metadata entries and quality check results.

Over the past few years, we have been continuously uploading and testing XAS data sets from different beamlines (different data formats and metadata structure) at the RefXAS database^5–7^ to examine the compatibility of interface with these data and file formats. As shown in the data workflow of RefXAS (Fig. 1), these uploaded datasets are first subjected to an automated quality control at the interface which is then followed by manual curation. Based on our experience with the curation of a significant number of XAS data/file formats uploaded at the database, the data quality can be estimated from two kinds of quality control. The first is *metadata quality control* where the metadata fields reporting basic and crucial information about the measured data are used to assess the main attributes of the data. The second is the *scientific quality control* where formulated quality criteria employing pre-processing or analysing of the data are used to estimate the useful information that can be extracted from it.

### Metadata quality control

The completeness of information in the metadata fields are a critical part of the initial quality control assessment as by reporting these fields one can already provide an overview of the usefulness or limitations of the data measured. For XAS in particular, the metadata fields in the present work have been classified into the sample description as well as the information about the instrument employed for measurement of XAS as follows:

### Sample

Describing the sample details initially is important, to start with the state of the sample during XAS measurement, i.e. solid/liquid as well crystalline/amorphous should be reported. Next step is to include details about sample preparation which can range from preparing simple *ex-situ* pellets to complex *in-situ*/operando reactors^8,9^. Sample preparation is crucial for getting good or measurable XAS signal specially in case of transmission mode^10^; the same concerns the use of the environmental cell^11^. The mass required to prepare a sample should be calculated in advance to get the optimized signal^12^. If the sample details are already properly documented digitally using available research data management (RDM) tool, i.e. electronic lab notebook (ELN)^13–15^, then it will make the tasks of ingestion into databases easy by importing that information directly from those ELNs and making it available to general users. This will additionally help users in identifying reference spectra best matching their respective experiment.

Sample identification in the field of XAS is currently not well established like other techniques, e.g., Crystallography, where well defined and documented sample metadata is available. In the field of XAS, there are different ways for identifying samples using specific identifier based on the application of identifier and there is no standard format to define the sample for an XAS experiment. In order to document the sample details, generally a short name or an identifier is used which can help in identifying the sample during different stages, i.e., synthesis, characterisation, etc. For example, while measuring a sample at any instrument, a short name is given to the file containing the data as well as metadata of the measurement, i.e. ‘collection code’. These collection codes in general are short names which often involve the chemical symbol of element/compound present in the sample and in some cases also the symbol of measured XAS edge (K, L, M etc.). In case of multiple scans of the sample, the scan number could be added as suffix to such already existing identifier. Such identifier helps in identifying the samples only among the people involved in the experiment or part of the small research group using similar collection codes. Thus, it has very limited reach. For documenting the sample details, there is always a possibility for users to mention the CAS (Chemical Abstracts Service) number^16^, if available, for identifying the standard chemical compounds. This however, is also only feasible for well-known standard samples which is not the case for XAS samples in general. In this direction, *Sample IDs* could provide a more unique identifier for each sample or process where any user can extract the details about the sample under investigation. It is very important that the samples, whose data are uploaded on any database, should be uniquely identifiable and the corresponding processing can be tracked^17,18^. Hence, a *sample ID* is required which is unique and persistent even though the samples themselves may not always be persistent. Other important metadata are temperature, pressure, sample environment and many other parameters which can impact the quality of the XAS spectrum. However, these parameters can also be part of the measurement process for *in-situ*/operando measurements in a cell or reactor. In the current form of metadata structure at RefXAS, we have included these external parameters under ‘Sample’. One of the options for extending metadata schema is to define a separate meta data field ‘Process (experiment)’ for detailing such kind of information related to experimental conditions as these *in-situ* cells/reactors are not in general part of or may be labelled external to the instrument, i.e. beamline, spectrometers employed for XAS data measurements. Thus, such metadata fields will not fit to the sub-field ‘Instrument’ discussed in section 2.2.

### Instrument

The settings or specifications of the instrument at which the XAS data has been measured form an important part of the metadata. Depending on the type of facility, i.e., whether synchrotron or laboratory, the metadata fields can be classified differently. In case of synchrotrons, beamline parameters such as acquisition mode, type of monochromator crystals, mirrors and detectors employed form the metadata part. Beamlines are state of the art instruments, which have been developed based on user-based applications and thus can operate under different detection modes, e.g., transmission, fluorescence, total electron yield. The mode in which the XAS spectra were measured will make it easy to understand the limitations of the involved acquisition method which in turn explains the spectral resolution, detected noise level, etc. Depending on the optics of the beamline and mode of measurement, different crystals are used such as DCM (Double Crystal Monochromator), CCM (Channel Cut Monochromator), Polychromator *etc*.^19^. Typically, crystal materials and their orientation are reported as metadata for any XAS experiments, e.g., Si(111), Ge(220), etc. Mirrors are also an important part of the beamline optics in order to filter higher harmonics as well for focussing/collimating X-ray beams. If a mirror is used then mirror (coating) material, incident angle and form of the mirror (plane, bent) are specified. Detectors, i.e., ionization chambers (IC), solid-state detectors (SDD) used to quantify the intensity of incident and transmitted (after sample) X-rays are part of the metadata thereby providing a complete overview. Other than the metadata fields for a beamline described above, we have provided a detailed list of metadata fields with short definitions for each field. We have reproduced this list here in Table 1 from ref. ^3^ for completeness of the discussion. These beamline details already provided here can be directly link to the spectra uploaded by the users, thereby making the process faster and easy. Using these metadata fields, an ingestion mask is made available at RefXAS interface for inclusion of a beamline so that such details are readily available to users while uploading their data. This additional beamline mask is currently made available only to developers and editor with database login credentials. Hence, a general user is not required to provide these detailed metadata for the beamlines while uploading the data at RefXAS, only metadata fields mentioned in normal upload mask of data are needed. The beamline details as given in Table 1 will be prepared by editor/curator of RefXAS database after getting them verified by the concerned beamline scientist or in best case can be provided by the beamline scientists themselves.

**Table 1:** Detailed list of the metadata fields for a Beamline. (reproduced from ref. ^3^ for completeness of the discussion).

Beamline metadata field	Description / Example
**Facility**	
Synchrotron name	identifies the specific synchrotron facility where the research is conducted, with corresponding PID (*The Research Organization Registry (ROR)* 2023)
Beamline	refers to the specific beamline used at the synchrotron, doi of description if available
**Storage ring**	
Energy	indicates the energy level of the electron beam in the storage ring. Unit: GeV
Beam emittance	describes the quality of the electron beam.Unit: nmrad
Filling mode	refers to the pattern in which electrons are distributed in the storage ring
**Mirrors**	
Use mirror	yes, no
Position	before / after monochromator, both
Reflecting surface material	specifies the material composition of the mirror’s reflecting surface.
Angle of incidence	refers to the angle at which the synchrotron light strikes the mirror. Unit: mrad
**Filters / windows**	
Filter	specifies the type and characteristics of the filter used in the beamline.
windows	describes the material and specifications of the windows in the beamline.
**Detectors**	
If IC used gases and pressure (IC: Ionization chamber)	separate fields for I0 and I1 (I0: Ionization chamber before the sample, I1: Ionization chamber after the sample)
If fluorescence detection	type of detection
**Scan parameter**	
Detection mode	identifies the method used for detecting the synchrotron radiation. e.g. Fluorescence, Transmission, electron yield
Scan mode	refers to the technique employed for scanning the sample with the beam. E.g. Continuous, Steps
Monochromatic flux on sample	indicates the intensity of the monochromatic beam as it interacts with the sample.
Beamsize on sample	specifies the size of the beam when it hits the sample.
Higher harmonic content of beam	actual, measured
**Source-type**	
Type	Undulator (tapered, scanned, …), Wiggler, Bending magnet
Critical energy	indicates the energy at which the intensity of the synchrotron radiation is at its peak. Unit: eV
Maximum k value	refers to the highest wavevector (k) value achievable. Integer (in Å^−1^)
**Monochromator**	
Type	design and functional type of the monochromator. e.g. DCM (Double crystal monochromator)
Crystals	e.g. Fe_2_O_3_ (hematite)DCM, CCM, Polychromator, specify materials and orientation, e.g. Si(111), Ge(220), etc.
Lattice spacing	indicates the spacing between atoms in the monochromator crystals. Used to calculate the energy axis
Temperature of crystals	refers to the operational temperature of the monochromator crystals. Unit: K
Distances: Source to DCM	e.g. (in Meters)
Distances: DCM to sample	e.g. (in Meters)
Encoder on theta axis	either No, or Yes - > Resolution (angle units)
Position of slits	distance from source or sample
Opening of slits	describes the aperture size of the slits in the monochromator setup
Energy resolution	indicates the precision with which the monochromator can select specific energy wavelengths of the X-ray beam
Detuning	refers to the deliberate misalignment of the monochromator crystals to reduce the intensity of higher-order harmonics. Unit: percentage of maximum intensity
**Beam damage (**If applicable**)**	
No	NaN
Yes	details

Similarly, if a laboratory XAS instrument is used then the metadata information required should consist of geometry of the spectrometer, i.e., von Hamos, Johan, Johansson^20,21^. If applicable, the name of the commercial instrument should be included as well as information on the source and X-ray tube’s anode material and operation parameters. Crystal material, thickness and their orientation, e.g., curved Highly Annealed Pyrolytic Graphite (HAPG) mosaic crystal or Spherically bent Bragg Si crystal, HAPG (002) or Si (111) along with diffraction order (“e.g.: 1”) and, if possible, bending radii can provide information about the process involved in the measurement, thus can be useful for estimating the maximum energy range available, detected noise level, etc. Depending upon the mode of detection, scanning or dispersive, detector type and mode should be included. i.e., SDD or position sensitive detector (CMOS, CCD, Hybrid-CMOS etc.). Similar to the synchrotron radiation facility-based spectrometers, the necessary metadata for laboratory-based instruments are highly depending on the specific instrument, especially the geometry. Based on the above discussed metadata fields for a laboratory XAS instrument, a separate mask has been made available at RefXAS database interface to upload the data measured at different laboratory XAS facilities. At RefXAS database, under the ‘Source’ field of general upload mask, one can select between ‘Synchrotron/Laboratory’ from the drop-down menu and based on the selection, the metadata fields shown in the ‘Instrument’ section will automatically switch to chosen category.

The metadata fields discussed above under the categories of sample and instrument which defines the specific XAS experiment, form the basis of metadata quality control for the ingestion of XAS data at the RefXAS database. In general, the details of these parameters can help to understand the outcome during the pre-processing or analysis of the data. More specifically, documenting these metadata parameters can provide other users with an overview of data attributes which will facilitate the FAIR use of XAS data. However, this metadata structure should not become the limiting factor for the data upload as it requires substantial effort from users, especially for beamline/instrument parameters. Within DAPHNE4NFDI we cross-link users, large-scale facilities, beamline scientists and IT-personal to find unified solutions for the meta data. We have further communicated with XAS beamline scientists across Europe to get an overview of the metadata saved in the raw data files using the macros available during beamtimes. Helpful in future would be if the beamline staff could implement automated parsers for common raw/metadata file formats. This could be achieved with less efforts once the beamlines switch to the NeXus data format for raw/processed data^22^; a task that then lies mainly in the hands of the large-scale facilities or the beamline scientists in general. Another issue to be resolved is that users are provided with process data files and not the raw data files in most of the cases which makes the availability of metadata limited. Thus, we have been trying to make users aware of the difference between raw and processed data (as discussed in next section) and also planning to get the metadata files directly from the beamline if made available by facilities.

### Scientific quality control

Before discussing *scientific quality control*, the difference between *raw data* and *processed data* for an XAS experiment should be clearly understood. *Raw data* obtained from different beamlines/laboratory spectrometers may in some cases only be machine readable and would need to be pre-processed before being imported into any legacy processing or analysis software. This raw form of the data has a well-defined format in most of the cases based on the instrument used and contains instrument settings details as metadata. Thus, this *raw data* needs to be processed using the scripts/macros developed by beamline scientist or manufacturer in some cases to convert it to processed form of the data, usually some kind of spectrum/spectra, which users can then import into the processing or analysis software. Hence, what users are provided with is somewhat the processed form of the data from their XAS measurements which can be referred to as *processed data*. For example, raw QEXAFS data from a beamline or images obtained from a laboratory spectrometer needs to be converted into a processed form, i.e., spectrum data with columns of E (Energy), I_0_ (Incident intensity), It (Transmitted intensity), µ (Absorption coefficient) which then can be further evaluated by a user or shared with other users. Hereafter, we will be referring to this *processed data* for defining the quality criteria and not the *raw data*.

**Fig. 2:**
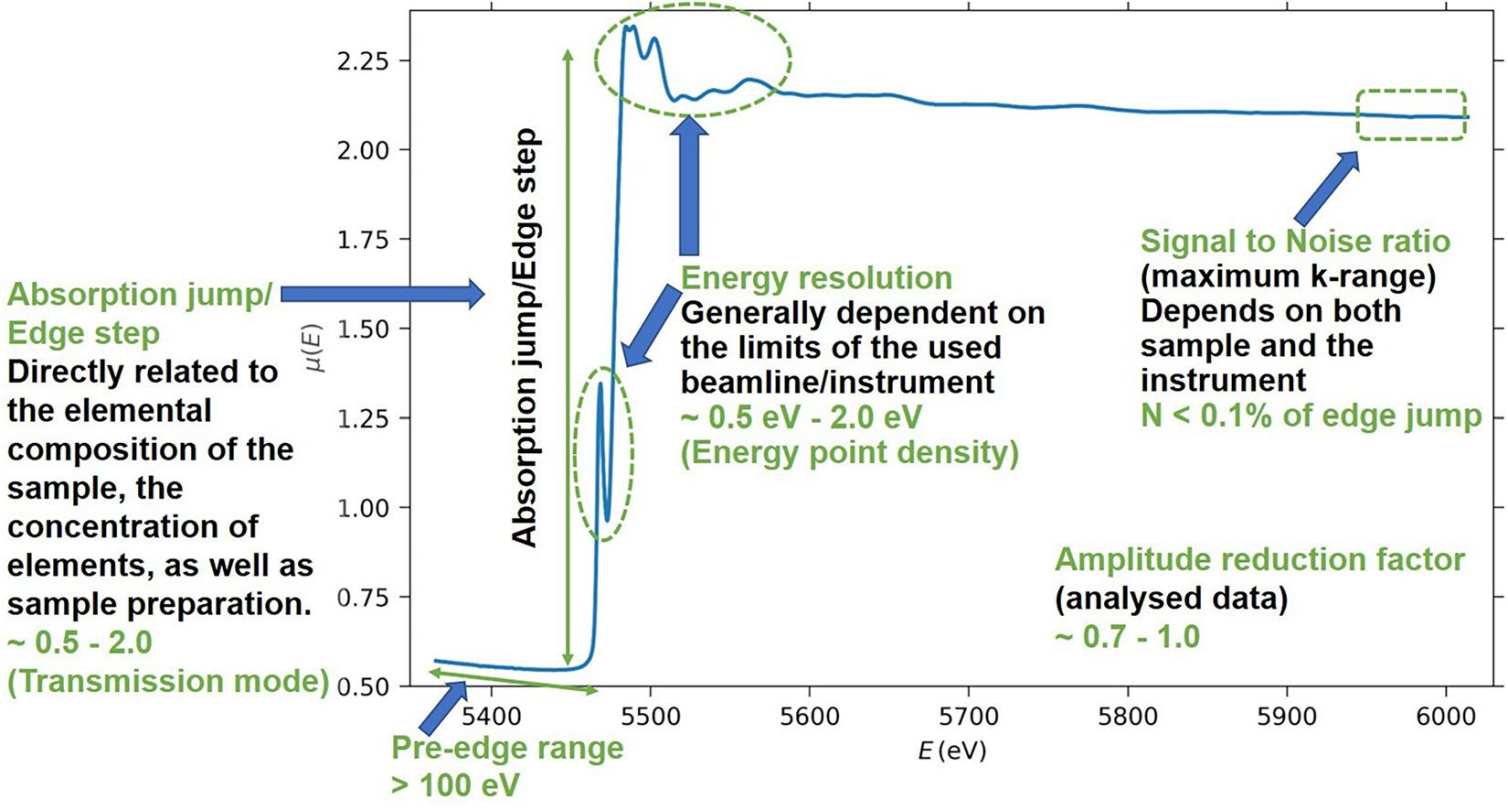
The depiction of scientific quality criteria in a typical XAS spectrum of Vanadium oxide at V K-edge and how they are correlated to the different regions of interest. For detailed definition of the parameters and their limits, please see the text.

Evaluating the quality of the measurement data scientifically, i.e., during pre-processing as well as the analysing is crucial to guarantee that the derived results are accurate and reliable. To start with, it is convenient to first define these *scientific quality criteria* for reference measurements, i.e., metal foils, oxides etc. Figure 2 shows some of these quality criteria for a standard XAS spectrum and how they are correlated to the different regions of interest in terms of XAS analysis. These parameters discussed below in detail are directly or indirectly related to the scientific outcome from the XAS data and hence are used for the initial screening of the XAS data uploaded at the RefXAS database:

#### Edge step (transmission mode)

As the incident X-ray energy reaches the absorption threshold or edge energy of an element, the higher probability of photoelectric absorption process gives rise to a steep curve, also referred to as onset of the absorption edge. So, there is a marked difference in absorption between energies before the edge and energies just after the edge. This difference in the absorption of the element at this edge energy is referred to as the *edge step* or *edge jump*. The X-ray attenuation coefficient μ(E) is correlated to the incident intensity (I_0_), the intensity of X-rays transmitted through the sample (I_t_) and the sample thickness (x) according to the Beer–Lambert’s law^23^. Hence, the *edge step* is directly proportional to the thickness of the sample for a given concentration of the element present in the studied sample. To be more precise, it is proportional to the number of absorber atoms in the beam path through the sample. The amplitude of the extended X-ray absorption fine structure, i.e., EXAFS oscillations obtained in the far energy region (50–1000 eV) is also proportional to the height of the edge jump. Thus, for the measurement of XAS for any sample an optimal edge step characterized by a steep onset of the absorption edge is preferred. It reduces background noise and influence of unwanted effects such as sample inhomogeneity, glitches etc. in the EXAFS data and improves the accuracy of the measurement results. But for too thick samples larger edge jump (more than 2 or 3 or higher) may indicate occurrence of undesirable effects such as e.g. influence of higher harmonics and/or non-linearity of detectors measuring very low photon flux after the sample. For some laboratory-based spectrometers this might be even more restricting as a highly asymmetric shaped spectrometer response function strongly influences EXAFS data quality i.e., dampening of the EXAFS oscillation amplitude, effectively shifting and decreasing the optimal edge jump interval^9,24,25^. Thus, *edge step* is an important indicator of the quality of the sample as well as the measurement. The preferred range of *edge step* for transmission measurements as suggested by different sources in literature is **0.5–2.0**^26–28^.

In case of too thick samples or samples with higher elemental concentration, the total absorption becomes dominant as compared to the edge jump. Total absorption includes absorption by all the material between the I_0_ and I_t_ detectors in addition to the sample, i.e. windows, substrates, air, etc. In such cases, the value of I_t_ should be checked before and after putting the sample in the beam and if there is a rapid fall by a factor of 20 or more, then sample is highly absorbing and not optimum for XAS measurement^26^. Thus, providing I_0_ and I_t_ values in the data column other than µ(E) for the XAS spectrum could give user an additional parameter to check for the signal quality.

**Fig. 3:**
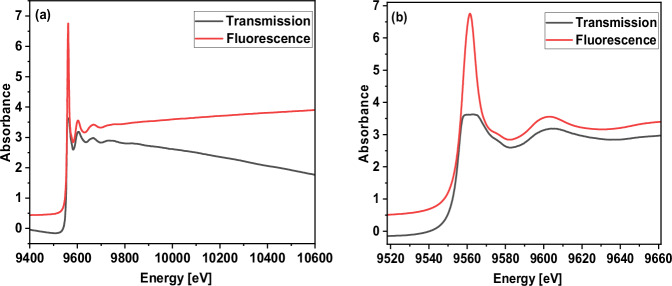
(**a**) As measured XAS spectra of a Hf-containing sample at Hf L_3_-edge measured in transmission and fluorescence mode showing effect of higher elemental concentration on the white line intensity and (**b**) Corresponding XANES region.

Figure 3 shows as measured XAS spectra of Hf-containing sample at Hf L_3_-edge measured in transmission and fluorescence mode at Beamline 10 at the 1.5 GeV Dortmund Electron Accelerator DELTA^29^. The spectrum measured in fluorescence looks fine with expected higher white line intensity due to the higher valency of Hf in the sample. However, for the spectrum measured in transmission strong dampening of the white line intensity is observed due to the thick sample, i.e., elemental concentration is not properly set before the measurement which caused strong dampening of the edge part in transmission mode^20,27^. The metadata for the XAS measurements is provided in Table 2.

**Table 2:** Metadata for XAS spectra of Hf containing sample.

**Sample:**
Sample: Li1.5HfCl4P0.5 powder
Physical State: Solid
Temperature: 298
Pressure: 1 bar
Sample Environment: Pellet in sealed, evacuated Al-bag
General Remarks: Sample provided by Meng-Fu Tu, Zhu Cheng, Marnix Wagemaker, Storage of
Electrochemical Energy, TU Delft, Mekelweg 15 2629 JB Delft, The Netherlands
Sample Preparation: Pellet pressed in a glove box under protecting atmosphere, ca. 20 mg without binder
**Instrument:**
Facility: DELTA, 1.5 GeV, 120–140 mA
Beamline: Beamline 10, Reference: Lützenkirchen-Hecht *et al*., 10.1107/S1600577514006705
Beam size: 0.35 mm (vertical) x 3.0 mm (horizontal)
Element: Hf
Edge: L3-edge
**Aquisition Mode: Transmission**
Crystals: Si111 Channel cut
Detectors: Two ionization chambers, each 15 cm long, N2-filled
**Aquisition Mode: Fluorescence**
Crystals: Si111 channel cut
Detectors: ionisation chamber, 15 cm long, N2-filled, for I0, PIPS-detector (50 mm diameter), for
fluorescence detection
**Processing:**
Calibration: Energy-calibration of sample spectrum with Zn Foil spectrum in Transmission.
Zn K-Edge observed at 9667.6 eV and calibrated to 9659.0 eV (E shift = 8.6 eV).
The energy scale of the Hf L_3_-edge spectrum is shifted accordingly with an E shift of 8.6 eV.
Method of Calibration: Using the first maximum in the first derivative of the absorption spectrum, i.e., µ(E)
Average/Merge: No (Single scan used for processing)
Truncation: No
Smoothing: No
Normalisation: No

#### Edge energy (calibration)

One of the first steps for screening of the measured XAS data is to check if the calibration is done correctly and the method of calibration used has been clearly mentioned. The absorption edge energy for all elements have been measured^30^ and are listed in X-ray periodic tables^31^. Experimentally, the edge energy (absorption threshold) can be defined as the first maximum in the first derivative of the absorption spectrum, i.e., µ(E) (for more definitions, please see^23,26^). In most of the XAS analysis tools, the smoothed derivative function is used to find the absorption edge energy, which is defined to be the maximum in the first derivative of the μ(E) spectrum. This method has proven useful for noisy spectra as the smoothing step makes detection of the derivative peak straightforward without compromising much on the resolution of the edge. Hence, this is also the default method used across the XAS databases^1,32^. This is the first step during evaluation of the measured XAS data and hence should be checked before proceeding further in processing the data. As for the standards or reference materials (e.g. foil) the value of edge-energy is known, thus in such cases during analysis of the data by user, calibration can be performed directly by calibrating the obtained edge-energy value (inflection point) to its postulated value. It is worth mentioning that this is the calibration performed by a user on the processed data and not to be confused with the calibration of the energy axis for raw XAS data obtained at a beamline where angle units are converted into corresponding energy units based on the Bragg equation^33^.

For a sample (like oxide) with a reference (foil) measured simultaneously, user should calibrate the reference first and apply the same calibration to the sample. The energy shift obtained during the calibrating of reference is applied to sample for the calibration of the sample data. This functionality has been incorporated in the recent developments in the RefXAS database using Larch functionalities^3,34,35^ and different formats provided by XAS beamlines to the users have been continuously tested at the interface. For samples with an unusual oxidation state for which there is no existing reference in the database, there is a possibility that errors may be made when curating and uploading such a spectrum to the database. The best way to verify the spectrum of such a sample is to check if the XAS results of such sample are already published or reported by user uploading the sample data or by previous workers in the field. However, the automated check is the first step, so we need to define how the scripts should respond in such cases where extremely large or small edge shifts are observed. Thus, defining quantitative thresholds, e.g., very high or negative edge shift, could provide possible solutions in such cases so that such spectra could be flagged during automated quality check.

The edge-energy (or edge-shift) provides important information about the oxidation state of the element studied. The absorption edge is known to shift towards higher or lower energy side relative to elemental edge, depending upon whether the absorbing atom bears positive or negative charge. The shift in the elemental edge of a sample is primarily influenced by the oxidation state of that element and a systematic shift towards the higher energy indicates an increase in the oxidation state. This correlation of edge-energies with oxidation state is a useful tool for the fingerprinting (speciation) methods employing X-ray absorption near edge structure (XANES^)36^. Considering the importance of edge-energies along with other XANES features, it has been proposed to add more metadata details as well as quality factors for extending the RefXAS database. For example, in case of first row transition metal edges, the pre-edge feature corresponds to the 1s → 3d transition and it is sensitive to the electronic structure of the metal atom site. The details of the energy position, splitting and intensity distribution of this pre-edge feature could provide information about the electronic structure in such cases. In addition, the edge shift of a sample with respect to the energy of the metal foil, position of the white line (first maximum) as well as presence of the shoulder feature in the edge could be added to get an overview of the electronic structure.

### Energy resolution

Energy resolutions for the detectors are usually given as full width at half maximum (FWHM). It could also be defined as the ability of a spectrometer to distinguish between two closely spaced X-ray photon energies. The phenomenon of XAS proceeds through the formation of a core-hole with a finite lifetime which corresponds to an uncertainty in energy thereby limiting the energy resolution. In the energy ranges commonly used for XAS, this lifetime broadening is of the order of a few electron volts. The resolution of the monochromator should be good enough to make the unavoidable lifetime broadening the limiting factor of spectral resolution.

**Fig. 4:**
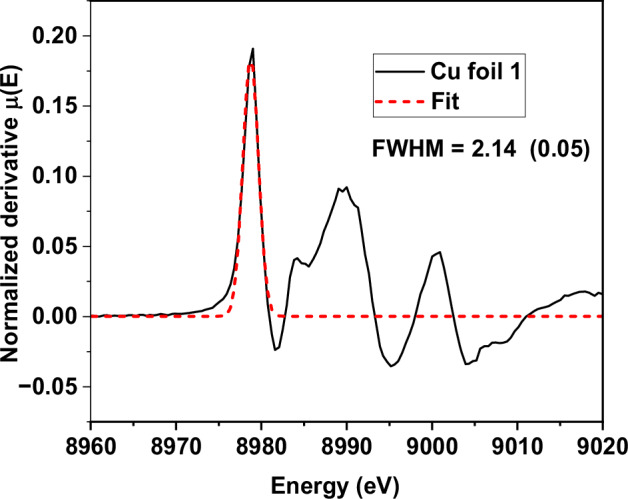
Estimation of the energy resolution by using the value of FWHM determined by fitting first maximum in the derivative µ(E) spectrum at the Cu K-edge for a metal foil. Gaussian model was employed for fitting the first derivative peak.

After the edge jump in the XAS spectrum, there are oscillations due to presence of neighbouring scattering atoms; these oscillations in general are referred to as fine structure. The amplitude of these fine structure oscillations can vary from one system to another, but in general it is ~10% of the edge jump in the near-edge region (XANES, 0–50 eV from edge) and down to ~ 0.1% or less for the extended-edge region (EXAFS, 50–1000 eV from edge)^37^. Thus, there are peaks/intense features in XANES whereas weak features of oscillatory nature occur in EXAFS. Energy resolution ranging between 0.5–2.0 eV could provide well resolved features in both XANES as well as EXAFS region. Based on the energy resolution, the energy step size through the edge must be chosen so that the features are well resolved. For more details on best practices for measuring XAS spectra, please see the recommendation by Meyer *et al*.^8^. As an example, the energy resolution for a Cu foil spectrum^38^ available at RefXAS has been determined using the FWHM of the first maximum in the derivative spectrum as shown in Fig. 4. The value as determined from this method gives energy resolution ~ 2 eV which is quite good resolution to measure XANES as well as EXAFS features in a Cu foil. In case of a sample spectrum by providing a reference spectrum, i.e., metal foil measured simultaneously, such estimation can be easily performed to determine the energy resolution. Note that, the resolution determined in this way is a convolution of the natural width of features in the spectrum (mainly due to the life time broadening) and not the resolution of the DCM in general. The resolution determined in this manner has contributions from the instrumental broadening, the core-hole lifetime broadening, and other intrinsic effects. At the RefXAS database, the automated quality check measures the energy step or point density as calculated from the spectral points of the XAS dataset uploaded. The value of energy resolution shown at the database refers to sampling frequency in the energy domain in the XANES region. We are currently developing/upgrading the different functionalities of the database based on more accurate measurement/definitions of quality criteria and the process of determining the energy resolution as shown here will be implemented in next developments.

### Maximum k-range (E-range) and SNR (Signal to noise ratio)

One of the limiting factors affecting processing of the XAFS data as well as results derived from the data analysis is available k-range (E-range). During processing of the XAFS data, a higher k-range can be very helpful in the proper normalisation of the spectra, i.e. post edge subtraction and then extraction of the fine structure part in E-space which is then transformed to k-space known as χ(k). Normalisation is a very crucial step before comparing the different XAS spectra in general whether processing time-resolved spectra for a sample or comparing sample spectrum with known references. Other than improving normalisation, the higher k-range, i.e., ≤15 could also be helpful to get well resolved peaks in R-space. The automated quality check results in the RefXAS database include a value of k-max which refers to the maximum usable value of k and not the maximum available value of k.

**Fig. 5:**
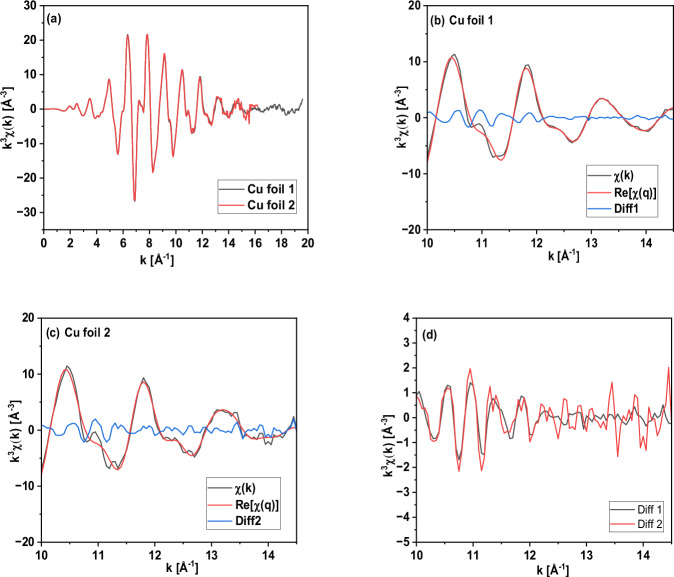
Determination of noise for Cu metal foil spectra using Fourier filtering method. (**a**) k^3^ χ(k) spectra for Cu metal foils, difference spectrum (noise) depicted in high k region for (**b**) Cu foil 1 and (**c**) Cu foil 2, and (**d**) comparison of noise obtained in each case. In Figures (**b**) and (**c**), the Re[χ(q)] represents the backward transformed data to q-space from R space by choosing the available R_max_.

Due to several factors involved, the SNR decreases effectively as we go to higher energies, i.e. as we go to higher k, the magnitude of χ(k) decreases. Thus, quality of an XAS spectrum can be considered good if the data is available to analyse till higher k-range, i.e., ≤ 15 and it has low noise, i.e., high SNR ratio. For the determination of noise, the Fourier filtering method can be used, which is also employed for smoothing of EXAFS data in some cases. In this method by Dent *et al*.^39^, after Fourier Transforming the EXAFS, i.e., χ(k) data into R space, by choosing the available R_max_, the data is backward transformed to q-space. Then, this smoothed χ(q) is subtracted from the experimental χ(k) data (using proper and same k-weight for both). The difference curve so obtained will provide an estimate for the noise in the spectrum. This method has been employed to compare the two XAS spectra as shown in the example below for the Cu foils, hdata taken from the RefXAS database^38,40^. Figure 5 depicts the different steps involved for determination of SNR using Fourier filtering methods. Table 3 provides the results of the statistics performed on the XAS data to determine SNR. At the RefXAS database this procedure for determination of noise is still to be implemented under automated quality check.

**Table 3:** Signal to noise ratio (SNR) determined for the two Cu foils, please see text for details.

Cu foil	Max (signal)	Min (signal)	RMS* (Noise)	SNR (max)	SNR (min)
1	21.63	−26.70	0.59	36.54	45.10
2	21.72	−26.41	0.82	26.31	32.00

*Root Mean Square

#### Amplitude reduction factor

S_0_^2^ is defined as the incomplete overlap between the passive electrons in the ground state and the final (correlated) ionic state of the system. It accounts for relaxation of the absorbing atom due to the presence of the empty core level and multi-electron excitations^23,37^. Though it seems only dependent on the absorbing atom properties, the bonding environment can also affect the value of S_0_^2^, thus its value may vary from one compound to another for an element. For the determination of S_0_^2^ a standard spectrum such as simple metal foil is measured in the same detector geometry and with the same beamline parameters as that of the unknown sample. S_0_^2^ is essential for accurately determining the coordination numbers from the EXAFS data analysis. It is typically obtained in the range of 0.7–1.0 and should not be higher than 1–1.05; values significantly outside this range should be critically looked at. In an extensive study, Kelly *et al*.^41^ showed that differences in beamline parameters and sample homogeneity could affect the value of S_0_^2^. Thus, its value is influenced by the settings of the beamline or spectrometer involved in the measurement and could provide a useful quality criterium as also reported in the recent results from an international round robin test for XAS spectra^42^.

### Sample-beam interaction (Beam damage)

In addition to the above-mentioned quality criteria affecting quality as well as usability of the measured XAS data, another critical factor to be mentioned is radiation or beam damage to the sample caused by beam-sample interaction. Beam damage may result from using highly focused intense beams, sensitive biological samples, or prolonged measurement times. Several factors can lead to beam-sample interactions and cause data artefacts. With the upgraded synchrotron sources, the high flux samples are getting exposed to may result in such interactions^43,44^. To control or minimise beam damage, one of the options is to move the sample so that different regions on the sample get exposed to the beam^8^. Also, by exposing some regions of the sample to the beam for longer times with multiple scan measured could help to determine the extent of beam damage. In such cases, it is very important to have uniformly prepared samples.

**Fig. 6:**
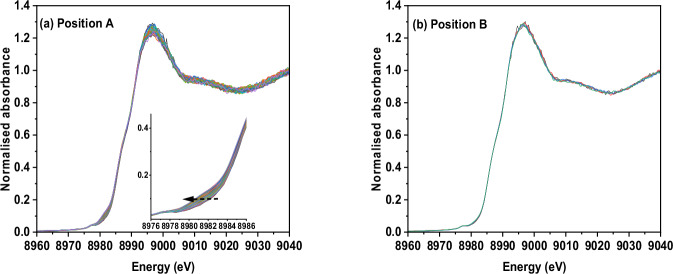
Cu K-edge XANES spectra of Cu based catalyst (**a**) at sample position A exposed to the focused beam for 10 mins, inset shows shifting of the edge indicating reduction in the zoomed-out energy region and (**b**) at sample position B exposed to the beam for 1 min.

Recently for evaluating the impact of the focused beam on the sample behaviour, Baumgarten *et al*.^45^ conducted beam damage test prior to the planned *in situ* and *operando* XAS measurements of a Cu based catalyst at the BALDER beamline at MAX IV Laboratory synchrotron facility in Sweden^46^. In a continuous flow capillary reactor filled with 10 mg catalyst diluted with SiO_2_, Cu K-edge XAS spectra were first measured for the sample position ‘A’ while exposing it to the focused beam for 10 minutes. The sample was then moved to get the beam on another sample position ‘B’ and spectra were measured for one minute. The XAS spectra measured at sample positions A and B at 120 °C under 5% H_2_ in N_2_ are shown in Fig. 6 (a) and (b), respectively. It was observed that at position A, the sample got reduced (almost 10%) even at low temperature (120 °C) which is mainly due to presence of focussed beam while the sample phase remains unchanged at position B. For more details of the sample preparation and XAS experiments please refer to the recently published study by Baumgarten *et al*.^45^.

We plan to include this beam damage parameter as quality criterion in the future for providing such critical details about sample-beam interaction at the RefXAS database. This can be a field like “Beam damage check performed: Yes/No” or a free comment field to describe the beam damage mitigation protocol by users.

### Fluorescence/HERFD-XAS

The fluorescence signal originating from a secondary process, measures the emission produced as a core hole is filled. Hence in this case the edge step is not related to decrease in the transmitted intensity or the elemental concentration^47^. Though similar metadata schema with some additional options for the detectors type etc., could be used in case of both transmission and fluorescence XAS spectra, the quality criteria applied or used in case of transmission mode XAS cannot be directly applied to those from fluorescence mode. The fluorescence spectra also need to be checked for the self-absorption of the emitted photon causing flattening of the signal or edge step resulting in distortion of the peaks or features in the XANES region. Advanced detection technique, such as high-energy resolution fluorescence detection (HERFD) is used for higher spectra resolution and to correct for self-absorption and obtain edge steps proportional to the true absorption coefficient. A HERFD-XAS experiment measures the intensity of the fluorescence line as a function of the incident energy (fluorescence-detected XAS, secondary- process detection) and may yield spectral features with line broadenings that are smaller than the lifetime broadening of the core-hole excited state of absorption.

Within the RefXAS initiative, we are working on formulating the quality criteria for uploading fluorescence as well as HERFD-XAS spectra. In case of HERFD-XAS, higher energy resolution can be achieved, however the metadata fields also need to be updated based on additional spectrometer details and geometry^48^. The following metadata fields have been under discussion:

**Table:** 

**HERFD-XAS: Metadata schema** for RefXAS database
Incoming X-rays: same fields as for the standard, including the measured edge.
Detection system / spectrometer:
Type of spectrometer
Radius
Number, type (e.g. Johann / Johansson) of crystals, reflection, comments (e.g. about crystal quality)
Angle of incidence
Detection system (e.g. APD, pixel detector etc.)
Energy resolution of the spectrometer in the current configuration (FHWM of the elastic peak)
Detection energy
Emission line (if applicable)

Note that reproducibility is much more demanding in this case as the geometry and instrument parameters are very decisive. HERFD-XAS, like standard fluorescence XAS, is affected by over-absorption. Therefore, the data should be checked and corrected for this distortion to distinguish spectral variations caused by experimental artefacts from the real spectral changes in the absorption coefficient^49^.

The further development of RefXAS database has been planned based on the above-mentioned points and to further increase the diversity of the compounds and the upload intake we have been in contact with more than 10 active XAS users’ groups interested in uploading of their reference data to the database. Next steps involved updating quality criteria for accommodating real samples as well as for uploading option for Fluorescence and HERFD XAS. In addition, upload functionality for the spectrum with a large amount of energy points, e.g., QEXAFS will be implemented.

## Discussion

Table 4 provides detailed description of Cu K-edge metal foil spectrum based on the metadata and scientific quality control parameters discussed in the previous sections. The metadata schema and automated scientific quality control results are taken from the RefXAS database website^38^ which are also part of the output files available to download from the interface.

**Table 4:** Detailed description of Cu K-edge metal foil spectrum based on the metadata structure and scientific quality control parameters formulated for uploaded datasets at the RefXAS database.

# *Sample Info*:
# - ID: PID.SAMPLE.PREFIX782d53bc-0b88-4c77-a02a-a9053c0568cd
# - Dataset Name: Cu-foil_K-edge_293K.dat 2024-11-27-14_33_50
# - Coll Code: Cu-foil_K-edge_293K
# - Physical State: Solid
# - Crystal Orientation: fcc
# - Temperature: 293 K
# - Pressure: 1 bar
# - Sample Environment: He flow cryostat
# - General Remarks: Metal foil
# - Sample Prep: High-purity commercial metal foil from Goodfellow Cambridge Ltd. with thickness of 5
μm and purity 99.97%
# - Sample Id: Link will be provided
# ----------------------------------------------------
# *Bibliography*:
# - Contact person: Alexei Kuzmin
# - Institute: Institute of Solid State Physics University of Latvia
# - Contact Email: a.kuzmin@cfi.lu.lv
# - 10.1002/pssa.202400623
# - Reference: Kuzmin, A., Dimitrijevs, V., Pudza, I. and Kalinko, A. (2024), Phys. Status Solidi A
2400623
# - Disclaimer: Yes
# ----------------------------------------------------
# *Instrument*:
# - Facility: DESY PETRA-III
# - Beamline: P65
# - Aquisition Mode: XAS Continuous mode
# - Crystals: Si111
# - Mirrors: M1-Si, M2-Si (both uncoated)
# - Detectors: Ionisation Chambers
# - Element Input: Cu
# - Edge Input: K
# - Max k Range: 19
# ----------------------------------------------------
# *Results quality control*:
# - Edge step: 1.235 a.u.
# - k-max: 19.7 Å^−1^
# - Energy resolution**: 2.0 eV
# - Edge energy: 8979 eV
# - SNR: SNR (max): 36.54
# - SNR: SNR (min): 45.10
# ----------------------------------------------------
# *Update / Verification Info*:
# (The following entries have been altered by Curator)
# - Updated/Verified by: Abhijeet Gaur, abhijeet.gaur@kit.edu, 27/11/2024

** Please see text for details.

The selection of each metadata field as provided in Table 4 is crucial for explaining the spectra uploaded to the database. A comprehensive set of metadata fields enhances the interpretation of XAS spectra, thereby improving its reusability and reproducibility. However, documenting every technical detail of a measured spectrum is not the solution. There should be a clear distinction between the requirements for raw and processed data, which facilitates the selection of appropriate metadata fields. As most databases provide data in processed form, the metadata schema should be designed to meet the requirements for processed datasets. Integration of a large language model (LLM) within the database which could significantly improve/semi-automate the upload procedure by providing dynamic guidance during the data upload process. An LLM could further help users by suggesting metadata fields, which would help in filling them and also ensure completeness and accuracy in the submissions, thereby reducing the likelihood of errors.

Other than documentation of critical metadata from XAS measurements, to complete the information loop for complex samples such as catalysts, battery material (electrodes) where details of the sample preparation as well as experimental conditions (*in situ*/operando) under which measurements were performed, also need to be documented. In such cases, Electronic Laboratory Notebooks (ELNs)^13–15,50^ are now becoming an essential part of FAIR (Findable, Accessible, Interoperable, Reusable) data management^51^. They document details of experiments, data collection, and analysis. ELNs help manage results collaboratively and link techniques to specimen information, crucial for understanding and reproducing experiments. The XAS databases should be integrated with ELNs for documenting complementary characterization. This integration will allow the inclusion or linkage of supplementary information on the sample characterisation, such as employing X-ray diffraction, Raman spectroscopy, UV/Vis, IR, etc. Integration of an ELN to the database will also be helpful to provide the link to the Sample IDs as pointed out earlier for tracking detailed information about the samples, e.g. synthesis and processes involved.

If the uploaded data at the database has been verified by experienced users/experts in the field and this protocol of quality checks is stated and agreed upon in the community, then this makes it reliable for the users to reuse it. Also, if the visualization tools are available at the database interface then users will be able to learn the processing/analysing steps involved . For example, in case of calibration of XAS spectra, there are different strategies depending on the element involved, the sample measured and acquisition mode (Transmission, Fluorescence, HERFD-XAS, etc.). Similarly, providing information on the different steps involved in the processing of the data, i.e., binning of spectral points, averaging of scans, normalisation, etc., could be very useful. In such cases, clearly mentioning the parameters chosen for such steps and further verification during curation will make it clear for any further reuse of the data. Thus, in this sense the role of curators/editors of the database is critical for development of database as a holistic tool.

In this context, formulating or reporting established quality criteria for manual/automated curation of the XAS data are crucial to guarantee that the derived results are accurate and reliable. Quality assessment is an important component to be included in the developing databases. Evaluation of the quality of the spectra (data) and metadata can ensure the accuracy and reliability of the data stored in the database, making it a valuable resource for the community.

However, the discussion on curation of data at XAS databases would be incomplete without considering the data/file formats. Standardisation of data formats in XAS community is an important step towards improving data usability, interoperability, and reproducibility^52,53^. In past as well as recent years, continuous steps have been taken within the XAS community for the development of standard formats^54^. The NeXus format^55,56^ provides a very good alternative for storing single/multiple XAS datasets as well as complementary measurements, i.e., temperature, gas analysis, etc., in a well-structured and self-explaining framework for data and related metadata. The NeXus format ensures that metadata is structured throughout and includes all the important parameters involved in the data acquisition. The metadata needs to be well-organized and comprehensive in order to get the accurate data predictions using ML algorithms. NeXus makes it possible that the structure of the metadata is consistent and complete as compared to other available data formats where crucial metadata is missing. The implications in opting for a particular format has been discussed in detail during the Q2XAFS workshops^52,53,57,58^. The availability of a suitable metadata schema in data formats used across the XAS community is critical and warrants further discussion. In general, ASCII is well suited to files containing a single type of spectrum, XDI gives a simple and very good way to represent a single XAS spectrum whereas HDF5 (NeXus) is preferable for datasets that combine multiple data types, such as XAS, gas-flow, and temperature data. There are other possible formats which include JSON, XML, and CIF. Much of this process could be automated using large language models.

## Conclusion

Data is considered reliable if it is reproducible and complete, and if it is free from truncation during processing from raw data to processed data, i.e., the conversion of raw data or file formats to processed data for analysis purposes. The process for obtaining the data should be clearly defined, with optimized equipment and documented measurement conditions. These qualities contribute to structured metadata and well-organized processing information for any measured data. One solution is to use the NeXus format for XAS data measured across different facilities. The integration of NeXus format with aforementioned quality criteria could provide a unique solution for both the deposition as well as the automated assessment of XAS data in databases.

Quality assessment of XAS data based on the discussed criteria could lay the groundwork for the further development of such practices, not only in XAS, but other techniques as well. Thoroughly evaluating the quality of spectra and metadata enables researchers to ensure the accuracy and reliability of their data, making it a valuable resource for the community.

## Supplementary information

Supplementary Information

## Data Availability

For the metadata schema implemented at RefXAS database and discussed in the present manuscript, a blank template in JSON format has been provided as a supplementary file to the manuscript. The metadata schema discussed and presented in this paper to share XAS data is also made publicly available in a machine-readable JSON, HDF5 and YAML format to the users upon downloading any selected spectrum from the database website http://xafsdb.ddns.net/. The different metadata fields and their accepted values have been discussed in detail in the present manuscript.

## Code availability

The custom code developed for the interface of the RefXAS database is also made available publicly at https://github.com/San-WierPa/xafsdb_webserver.
